# Low-temperature vacuum evaporation as a novel dehydration process for the long-term preservation of transplantable human corneal tissue

**DOI:** 10.1007/s10561-024-10155-y

**Published:** 2025-01-09

**Authors:** Owen D. McIntosh, Emily R. Britchford, Lydia J. Beeken, Andrew Hopkinson, Laura E. Sidney

**Affiliations:** 1 Academic Ophthalmology, Mental Health and Clinical Neurosciences, School of Medicine, University of Nottingham, Nottingham, UK; 2 NuVision Biotherapies Ltd, Medicity, Nottingham, UK; 3 Regenerating and Modelling Tissues, Translational Medical Sciences, School of Medicine, University of Nottingham, Nottingham, UK

**Keywords:** Cornea, Transplant preservation, Lyophilisation, Cryopreservation, Tissue processing

## Abstract

Globally there is a shortage of available donor corneas with only 1 cornea available for every 70 needed. A large limitation to corneal transplant surgery is access to quality donor tissue due to inadequate eye donation services and infrastructure in many countries, compounded by the fact that there are few available long-term storage solutions for effectively preserving spare donor corneas collected in countries with a surplus. In this study, we describe a novel technology termed low-temperature vacuum evaporation (LTVE) that can effectively dry-preserve surplus donor corneal tissue, allowing it to be stored for approximately 5 years, shipped at room temperature, and stored on hospital shelves before rehydration prior to ophthalmic surgery. The dry-preserved corneas demonstrate equivalent biological characteristics to non-dried donor tissue, with the exception that epithelial and endothelial cells are removed and keratocytes are rendered non-viable and encapsulated within the preserved extracellular matrix. Structure and composition of the dried and rehydrated corneas remained identical to that of non-dried control corneas. Matrix-bound cytokines and growth factors were not affected by the drying and rehydration of the corneas. The ability to preserve human donor corneas using LTVE will have considerable impact on global corneal supply; utilisation of preserved corneas in lamellar keratoplasties, corneal perforations, ulcers, and tectonic support, will allow non-preserved donor tissue to be reserved for where it is truly required.

## Introduction

Globally there is a significant population of patients suffering from the health, social and economic issues associated with corneal blindness. Worldwide, only 185,000 corneal transplants are performed annually, despite the fact that there are 1.5 million cases of corneal blindness diagnosed each year (Machin et al. 2020) (Gain et al. 2016). Corneal transplant is the mainstay of treatment for restoring visual function, however limited access to domestic eye banks presents a major limitation in meeting the worldwide demand for donor corneas, with only 1 cornea available for every 70 needed (Gain et al. 2016). The additional impact of the Sars-Cov-2 pandemic has also led to high levels of tissue rejection from eye banks (Ang et al. 2020), in addition to reduced routine surgery.

One of the biggest global challenges to supplying transplantable corneas is access to quality donor tissue and inadequate eye donation services and infrastructure. This is compounded by the fact that there are limited effective long-term storage solutions for efficiently preserving donor corneas collected in countries with a surplus. Lack of standardisation in current eye banking methodology exists at national and international levels, with eye banks predominantly choosing to employ either hypothermic storage of grafts between 2 and 8 °C for up to 14 days, or organ culture which maintains viable corneas at 31–34 °C for up to 28 days (Armitage 2011; Pels 1997). Exporting corneas is challenging due to the need for controlled temperatures during shipping: including the need for electronic devices on airplanes to monitor temperature and financial limitations of temperature-controlled storage at the receiving end (Oliva et al. 2019; Wojcik et al. 2021).

When performing traditional full-thickness corneal transplants (penetrating keratoplasty, PK), endothelial cell viability is required to maintain of corneal clarity (Wojcik et al. 2021). However, the development of new surgical techniques including deep anterior lamellar keratoplasty (DALK) (Manche et al. 1999; Zaki et al. 2015), superficial lamellar keratoplasty (SALK) (Ganger et al. 2016), and lenticule insertion surgeries (Lim et al. 2013; Moshirfar et al. 2018), where the host’s endothelium can remain intact, has revolutionised corneal transplantation leading to eye banks evolving their services to provide pre-cut corneal tissue for partial thickness grafts. Pre-cut corneal tissue without epithelial and endothelial layers can therefore be provided for performing stromal lamellar surgeries or partial thickness grafts. Partial-thickness transplantation is currently utilised for a plethora of indications, including keratoconus, pellucid marginal degeneration, stromal dystrophies, stromal scarring and ulcers, infectious keratitis and emergency tectonic issues (Karimian et al., 2010), demonstrating a high safety profile, reduced risk of rejection, and efficacious visual results (Keenan et al. 2012).

These surgical advancements have spurred the development of term storage of corneal tissue preservation methods, aimed at maintaining the epithelial and stromal extracellular matrix (ECM) integrity (Chaurasia et al. 2020). This approach offers surgeons with an equivalent to traditional donor tissue, ensuring comparable visual outcomes and corneal clarity. Additionally, it facilitates easier global transportation and the potential for maintaining corneal graft stocks in hospitals. Increasing human donor corneal tissue longevity would not only ensure the availability of graft tissue in emergency situations but would also provide a clear solution to mitigate the disparate tissue supplies worldwide. It would allow for straightforward exportation from countries with a surplus of corneal graft tissue, whilst decreasing waste and increasing available transplant tissue, through utilisation of corneas which have surpassed their original storage time.

Current methods in development for the long-term preservation of human donor tissue include cryopreservation (Eastcott et al. 1954; Halberstadt et al. 2003), decellularisation (Lynch et al., 2013; Rovere et al. 2019), irradiation (Thirunavukarasu et al. 2023) and dehydration technologies (M. R. Feilmeier et al. 2010), alone or in combination. In many cases of tissue preservation, the natural cornea structure and characteristics are damaged, potentially causing complications post-transplantation such as oedema, swelling and opacification (Chaurasia et al. 2020).

We previously developed a drying process for human amniotic membrane that does not feature a pre-freeze step and effectively preserves the biological structure, function and composition of the tissue (C. L. Allen et al. 2013; Hopkinson et al. 2020; Marsit et al. 2019).In this study, we report using this novel dry-preservation technique designated low-temperature vacuum evaporation (LTVE) to dry-preserve human donor corneas. The LTVE process uses application of a vacuum, via a pump in a freeze-dryer, causing varying low pressures to modulate the boiling point of water, essentially speeding up the evaporation of water from the tissue. This occurs because less energy is required to expand the liquid molecules into a gas, so a lower temperature is required. Lowering the pressure also has the effect of increasing the freezing point of water, so the pressures and temperatures applied vacuum need to be carefully balanced to ensure this does not happen to the tissue and cause freeze damage. Thus, in our method the pressure is not allowed to go below 1.5 mbar by turning the vacuum pump on and off at set intervals. Overall, the process allows for delicate and effective tissue dehydration via evaporation without the addition of any chemicals, freeze steps or excessive heat. We investigate the effects of drying by LTVE and subsequent rehydration on the structural and biological properties of human donor corneal tissue.

## Methods

### Materials

Reagents were purchased from Thermo Fisher Scientific, UK unless otherwise stated.

### Human tissue

Anonymised human corneas were obtained from SightLife (now CorneaGen, WA, USA) under a materials transfer agreement. All work was performed in a laboratory under a research license from the Human Tissue Authority, UK. Informed consent was obtained from donors/relative prior to collection. All experimental corneas were delivered and stored in Optisol GS (Bausch + Lomb, NJ, USA) and stored at 4 °C until use. Corneas were 14—28 days from recovery.

### Preparation of human corneas for drying

Corneal buttons were prepared by punching out the middle of a cornea using a sterile 8.5 mm trephine. Control corneas (non-dried static controls) were immediately returned to the Optisol medium and kept static at 4 °C until the LTVE process for the experimental corneas had completed (approximately 8 h) and all corneas were processed for onward analysis. Agitated control corneal buttons (non-dried agitated controls) were transferred to Phosphate Buffered Saline (PBS); 5% (w/v) dextran 70 (Sigma-Aldrich, Dorset, UK) and 100 mM raffinose pentahydrate and went through the agitation step but were not dried. Corneal buttons for drying were transferred aseptically to a scintillation vial containing the sterile agitation media of either: PBS alone; 5% (w/v) dextran 70 and 100 mM raffinose pentahydrate in PBS; or 5% dextran and 100 mM raffinose pentahydrate with 1 mg/mL epigallocatechin gallate (EGCG, Sigma-Aldrich) in PBS. Scintillation vials containing corneal buttons were agitated on a rotator (Grant PTR-60) at 60 rpm for 60 min at room temperature.

PBS was used as an isotonic solution similar to saline. Dextran was used to make an iso-osmotic solution, to prevent movement of water into the cornea causing swelling; it is a commonly used addition to corneal organ culture medium, such as Optisol (Lindstrom et al. 1992; Lynch et al. 2016; Peyman et al. 1979). Raffinose is a complex saccharide used as a protectant during drying; we have previously published that raffinose is a potent protector of structure when drying amniotic membrane using a technique similar to LTVE (C. L. Allen et al. 2013; Marsit et al. 2019). The anti-oxidant EGCG has previously been shown to promote the viability of cells following freeze drying and was added as a cell protectant (Natan et al. 2009).

### Low-temperature vacuum evaporation preservation of human corneas

Post-agitation, corneal samples were aseptically transferred to 10 mL glass vials (Schott via Adelphi Healthcare Packaging, West Sussex, UK) and a bromobutyl lyophilisation stopper (West Pharmaceutical Services via Adelphi) placed loosely on each vial. Vials were transferred to a FreeZone Stoppering Tray Dryer with Freezone 6L Drying Console (Labconco, Kansas City, MO, USA) and dried at a shelf temperature of 25 °C and condenser temp -55 °C. To reduce the pressure but avoid freezing, the vacuum pump was switched on for 2 min, reducing the chamber pressure from atmospheric pressure to approximately 1.5 mbar before being switched off for 5 min. To fully dry the corneal tissue, 36 cycles of pump on/pump off were performed taking 180 min. Once dried, the pressure in the system was returned to atmospheric and vial stoppers pushed down and aluminium flip-up, tear-off crimp seal applied (West via Adelphi). Rehydration of corneal buttons was performed by injecting 3 mL sterile rehydration solution (5% (w/v) dextran in 0.9% (w/v) NaCl) into the vial.

Using a freeze dryer such as the one described allows up to 200 corneas to be dried at the same time. For all individual experiments, the corneas were dried together to minimise any variation of drying. For each experimental group 5 different corneal donors were used.

### Measurement of corneal weight and thickness

Corneal weights (pre-drying, dry, and rehydrated) were taken using an Ohaus Adventurer Balance. Weights were taken prior to experimentation start after storage in Optisol GS at 4 °C for up to 28 days. Corneal buttons were placed on pre-weighed weigh boats and the weight of the weigh boats subtracted. Cornea thickness was measured using digital callipers; 3 central thicknesses were taken per corneal button at each timepoint and averaged. Initial weight and thickness were taken immediately out of Optisol storage medium prior to agitation, drying and rehydration. Subsequent weights and thicknesses were compared to the initial weight.

### Transparency measurement

Baseline transparency readings of the corneas were taken immediately out of the Optisol solution. Non-dried static controls were placed back into Optisol, and all others were placed the relevant agitation solution before being dried, with the exception of the non-dried agitated controls. Transparency readings were then taken again once dried and rehydrated or after storage for the same amount of time for non-dried controls. Light transmittance through the corneas was measured using a CLARIOstar plate reader (BMG LABTECH, Buckinghamshire, UK) at 492 nm with 12 readings taken across each corneal button and averaged. Transparency was compared to the initial reading of the same cornea to account for biological differences in corneal transparency not caused by drying.

### Metabolic activity assay

Metabolic activity was measured using PrestoBlue™ Cell Viability Reagent (Invitrogen, ThermoFisher, UK). Samples were placed in a 24-well plate and covered in 10% (v/v) Presto Blue reagent in Hank’s Balanced Salt Solution (HBSS, Gibco, ThermoFisher). The plate was immediately transferred to a CLARIOstar plate reader pre-set at 37 °C and fluorescence readings at excitation 560 nm/emission 590 nm were taken every 30 min for 150 min.

### LDH release assay

The Pierce lactate dehydrogenase (LDH) assay kit was used to quantify levels of LDH released into the rehydration media of dried corneal buttons, to estimate levels of cell membrane lysis. The assay was performed according to the manufacturer’s protocol. Briefly, 50 µL of rehydration media and 50 µL of reaction mix were transferred to a 96-well plate and incubated at room temperature for 30 min. The optical absorbance was read on the plate reader at 490 nm with background correction at 690 nm. The maximum levels of LDH that could be released from a corneal button was assessed using non-dried corneal buttons that had been agitated in 1% (v/v) sodium dodecyl sulphate detergent at 37 °C for 24 h.

### Histology

Samples were fixed in 4% paraformaldehyde overnight, before washing and storage in PBS. Samples were prepared for sectioning in a Tissue Processor (Leica TP1020) through a series of graded ethanol solutions, then paraffin embedded. Sections (7 µm) were cut using a Leica 2245 microtome and transferred to adherent glass slides (SuperFrost Plus, ThermoScientific). Samples were de-paraffinised in xylene and rehydrated in a series a graded ethanol solution. Regressive haematoxylin and eosin staining was performed using Harris Haematoxylin and 1% eosin. Alcian blue and fast red staining was performed to visualise acid mucosubstances and red cell nuclei. Slides were mounted in DPX after staining and imaging was performed on a Leica DM1000 upright microscope with an MC170 Camera.

### Hydroxyproline assay

Hydroxyproline assays were performed as described previously (Edwards et al., 1980; Sidney et al., 2018) to estimate the levels of collagen within the corneas. Corneal buttons were digested in a 0.1 mg/mL papain solution in 0.2 M sodium phosphate buffer containing 8 mg/mL sodium acetate, 4 mg/mL ethylenediaminetetraacetic acid, and 0.8 mg/mL L-cysteine hydrochloride agitated at 65 °C overnight. Briefly, acid hydrolysis of papain digested samples was achieved by heating samples with concentrated hydrochloric acid to 120 °C for 5 h. Subsequently, samples were dried at 80 °C until only residue remained, which was dissolved in 0.2 M sodium phosphate buffer. Samples were transferred in triplicate to a 96-well plate, an equal volume of 70 mM chloramine T solution was added and incubated at room temperature for 20 min. Subsequently, an equal volume of 1.16 M dimethylaminobenzaldehyde solution was added and samples incubated at 60 °C for 30 min. Colour change was assessed by absorbance at 540 nm. Hydroxyproline concentration was calculated using a standard curve. Collagen concentration was estimated using a conversion factor of 7.6. Collagen readings were corrected for the original weight of the corneal button.

### Sulphated glycosaminoglycan (sGAG) assay

Corneal buttons were digested in papain as described above. The Blyscan™ 1,9 dimethyl methylene blue (DMMB) assay (Biocolor Ltd., Belfast, UK) was performed on samples according to manufacturer’s instructions. Briefly, 200 μL of papain digest was added to 1 mL DMMB dye solution and agitated for 30 min, before centrifugation at 10,000 × g for 10 min. The pellet was dissolved in 0.5 mL dissociation reagent and 200 μL transferred to each well of a 96-well plate. Absorbance was measured at 656 nm. sGAG concentration was determined using a standard curve. sGAG readings were corrected for original weight of the corneal button.

### Fluorescent immunohistochemistry

Corneal buttons were paraffin-embedded and sectioned as for histology (detailed above). Samples were deparaffinised in xylene and rehydrated through a series of graded ethanol solutions. Antigen retrieval was performed in a pH 6.0 sodium citrate buffer (Vector) at 95 °C for 60 min. Sections were permeabilised in 0.1% (*v*/*v*) Triton-X100 for 10 min and subsequently washed three times for 5 min in PBS. Non-specific protein binding was blocked using a solution of PBS with 1% bovine serum albumin (BSA), 0.3 M glycine and 3% (*v/v*) donkey serum, for 1 h at room temperature. Primary antibodies were diluted in PBS containing 1% BSA and 0.3 M glycine as follows: polyclonal mouse anti-Collagen-I (Sigma-Aldrich, dilution 1:200) and polyclonal rabbit anti-laminin (Millipore, dilution 1:100). Sections were incubated with the primary antibodies for 1 h at room temperature before washing three times in PBS. Either donkey anti-mouse Alexa-Fluor 594 or donkey anti-rabbit Alexa Fluor 488 secondary antibodies (Life Technologies, dilution 1:300) were applied to the samples at room temperature for 1 h. Samples were rinsed in PBS three times and counterstained with 4’,6-diamidino-2-phenylindole (DAPI; 1:200,000, Life Technologies). Samples were mounted in fluorescent mounting medium (Dako, UK) and imaged using a Leica DMIL LED inverted microscope with a Leica DFC camera.

### Transmission electron microscopy

Samples were fixed in 3% (v/v) glutaraldehyde in sodium cacodylate buffer solution (0.2 M, pH 7.2) for 24 h at 4 °C. Corneal buttons were post-fixed with 1% osmium tetroxide in sodium cacodylate buffer solution for 2 h at room temperature, followed by washing in sodium cacodylate buffer (0.1 M, pH 7.2), serial dehydrations in ethanol, and washing in propylene oxide (TAAB Laboratories Equipment Ltd). The corneas were embedded in araldite resin (TAAB Laboratories Equipment Ltd) and sectioned (Leica EM UC6; Leica Biosystems). Ultrathin sections of 90 nm were contrasted with uranyl acetate and lead citrate and observed on a transmission electron microscope (TEM, FEI Tecnai Biotwin T12), operating at 100 kV. Images were taken using a SIS Megaview digital camera (Olympus).

### Enzyme-linked immunosorbent assays (ELISAs)

Dried corneal buttons were stored under vacuum at room temperature for 6 months and compared to donor cornea in Optisol, stored for up to 1 month. Samples were chopped into small pieces using a scalpel no. 22. Tissue was transferred to a microcentrifuge tube in IP Lysis Buffer (ThermoFisherScientific, UK) and homogenised using an electric homogeniser. Homogenised samples were stored at -80 °C before analysis**.** Human DuoSet ELISAs (R&D Systems, Abingdon, UK) were used in combination with the appropriate DuoSet Ancillary Reagent Kit (R&D Systems), according to manufacturer’s instructions, to detect protein levels in the homogenised sample. Optical density at 450 nm with background correction at 540 nm was determined immediately after the addition of the stop solution. Protein concentration was determined using 4-parameter fit standard. Data was corrected for original weight of the samples to normalise for different sized samples.

### Statistical analysis

Statistical significances were analysed using GraphPad Prism version 9.3.1. Comparisons between multiple groups were compared using two-way ANOVA with post-hoc Tukey’s multiple comparison test and comparisons between two groups (ELISA data) were performed using unpaired Student’s t-test.

## Results

### Effect of dry-preservation on physical and biological characteristics of corneal buttons

Human corneal buttons mechanically agitated in a range of media prior to dry-preservation and subsequent rehydration. The corneal buttons were not pre-frozen before low pressures were applied and the process of drying did not cause any freezing. Average weight, thickness and representative transparency bitmap image for corneas at the start of the experiment (before any treatment) and end of the process (after storage for controls or drying and rehydration for experimental samples) can be seen in Table 1.

**Table 1:** Average storage time, weight, thickness and representative transparency image before and after drying

Group	Mean Storage Time^b^ (days)	Weight (mg)			Thickness (µm)			Transparency (OD)^a^		
		Start^c^	End^d^	%Change^e^	Start^c^	End^d^	%Change^e^	Start^c^	End^d^	%Change^e^
Non-Dried Static Control	23.0 (n = 5)	105.34 ± 11.88	109.92 ± 18.12	104.2 ± 11.9	792 ± 60.16	772 ± 24.90	98.0 ± 9.4	0.78 ± 0.07	0.80 ± 0.05	102.4 ± 12.9
Non-Dried Agitated Control	24.6 (n = 5)	107.32 ± 11.50	112.98 ±19.11	105.6 ± 17.7	802 ± 43.24	794 ± 20.73	99.9 ± 6.6	0.78 ± 0.06	0.80 ± 0.09	102.0 ± 6.7
Dried—PBS	22.0 (n = 5)	116.34 ± 14.30	116.54 ± 10.93	100.5 ± 12.7	816 ± 96.85	1004 ± 37.81	124.1 ± 11.1	0.77 ± 0.07	0.97 ± 0.11	125.0 ± 14.0
Dried —Dextran/ Raffinose	23.2 (n = 5)	121.52 ± 22.55	122.34 ± 30.77	99.9 ± 12.2	806 ± 89.61	822 ± 47.12	103.4 ± 16.9	0.72 ± 0.04	0.75 ± 0.07	104.1 ± 9.0
Dried—EGCG	23.8 (n = 5)	111.10 ± 17.64	109.80 ± 24.76	98.5 ± 11.0	802 ± 45.50	± 53.85	102.8 ± 12.1	0.71 ± 0.18	1.19 ± 0.32	167.8 ± 17.1

^a^Representative well scan transparency image taken via plate reader (green = more transparent; red = more opaque). Transparency reading at 492 nm given in optical density

^b^Mean number of days of storage in Optisol GS after retrieval before use in experiments

^c^Start—Weight/thickness/transparency prior to agitation, drying and rehydration

^d^End—Weight/thickness/transparency after agitation, drying and rehydration

^e^Percentage change between start and end readings for weight, thickness and transparency

**Fig. 1:**
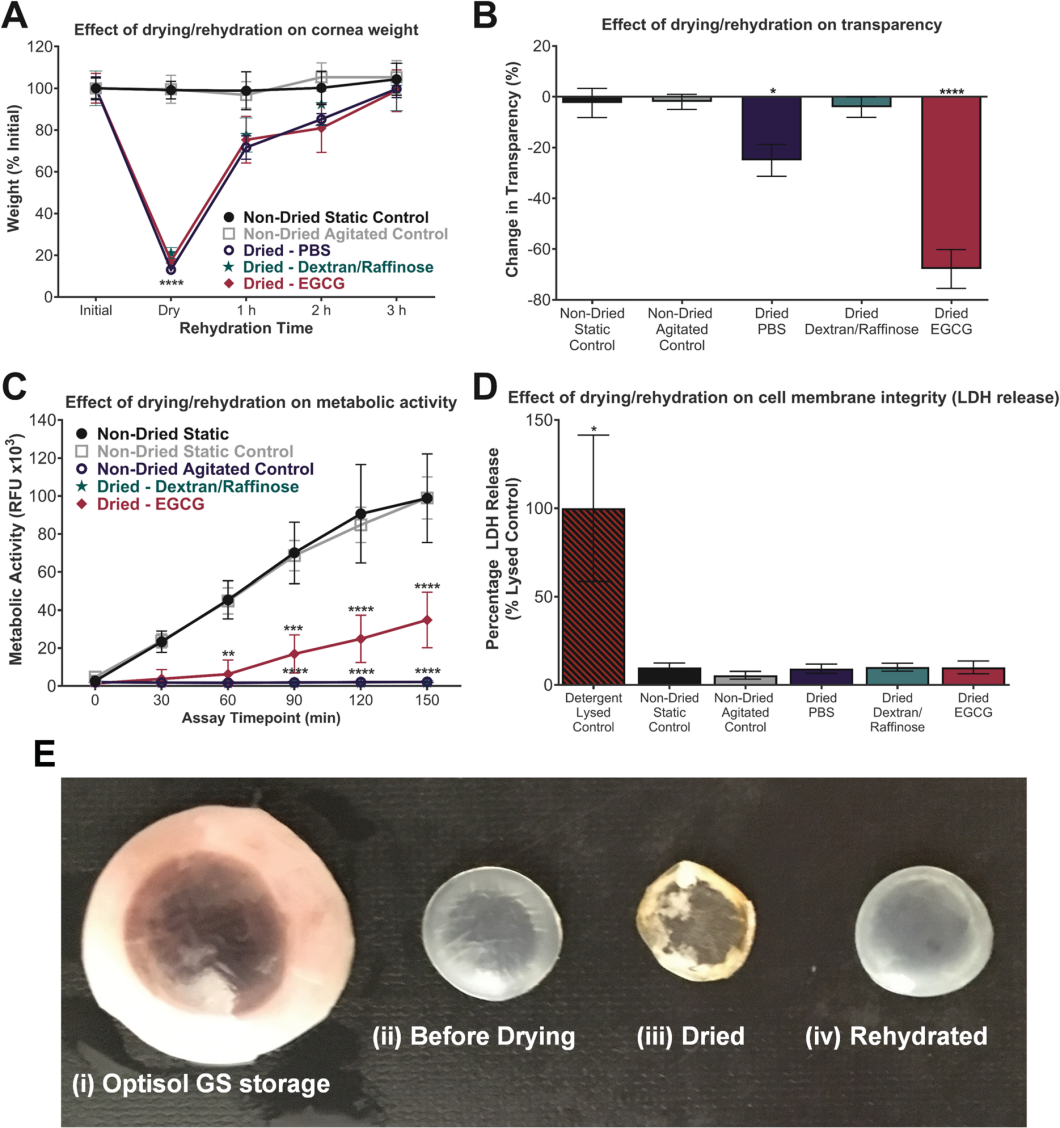
Effect of dry-preservation on weight, transparency and metabolic activity of human corneal buttons. Human corneal buttons were dried after agitation in PBS, 5% dextran or 5% dextran with EGCG and compared to non-dried controls that had remained static or were agitated. Dried corneal buttons were rehydrated in NaCl with 5% (w/v) dextran. **A** Change in weight of corneal buttons upon drying and rehydration for 3 h. Data displayed as % of initial weight, **B** Change in transparency of corneal buttons after drying and rehydration. Data shown as percentage change in transparency from initial weight, **C** Metabolic activity of cells within corneal buttons measured over time after drying and rehydration, **D** LDH release from sample after rehydration versus as a percentage of the SDS-lysed control. Data for A-D represented by mean ± SEM (n = 5). Statistical significance vs. non-dried static control: * p ≤ 0.05, **p ≤ 0.01, ***p ≤ 0.001, ****p ≤ 0.0001. **E** Images of (i) Optisol-stored cornea prior to drying, (ii) 8.5 mm corneal button before drying, (iii) corneal button after drying, (iv) corneal button after drying and rehydration

Dehydration and subsequent rehydration did not change the rehydrated weight of the corneal tissue (Fig. 1A). The dried weight, regardless of agitation media, was shown to be approximately 20% of initial weight with rehydration returning the corneal buttons back to original weight within 3 h. Transparency was measured quantitatively by plate reader optical absorbance assessment (Fig. 1B). The transparency level of corneal buttons agitated with dextran was similar to that of the non-dried static (Optisol -stored) and agitated controls. However, PBS or EGCG pre-treated corneas showed a significant negative effect on corneal transparency. Metabolic analysis of the corneal buttons subjected to different agitation media prior to drying and rehydration led to the elimination of metabolic activity in the tissue when compared to non-dried controls (Fig. 1C). Pre-treatment with EGCG allowed some cell viability to be maintained despite the drying process, however when treated with PBS or dextran this activity was lost. Non-dried controls were shown to retain significant cell viability. Cell membrane rupture of keratocytes within corneal buttons was determined by measuring the level of LDH released into the rehydration media (Fig. 1D). There was no difference in LDH released in dried verses non-dried control corneas following rehydration. This was compared to SDS-lysed controls which showed significant rupture and increase in LDH activity. Corneal buttons after drying and rehydration were macroscopically comparable to non-dried controls (Fig. 1E).

### Effect of dry-preservation on collagen and glycosaminoglycan structure of human corneal buttons

**Fig. 2:**
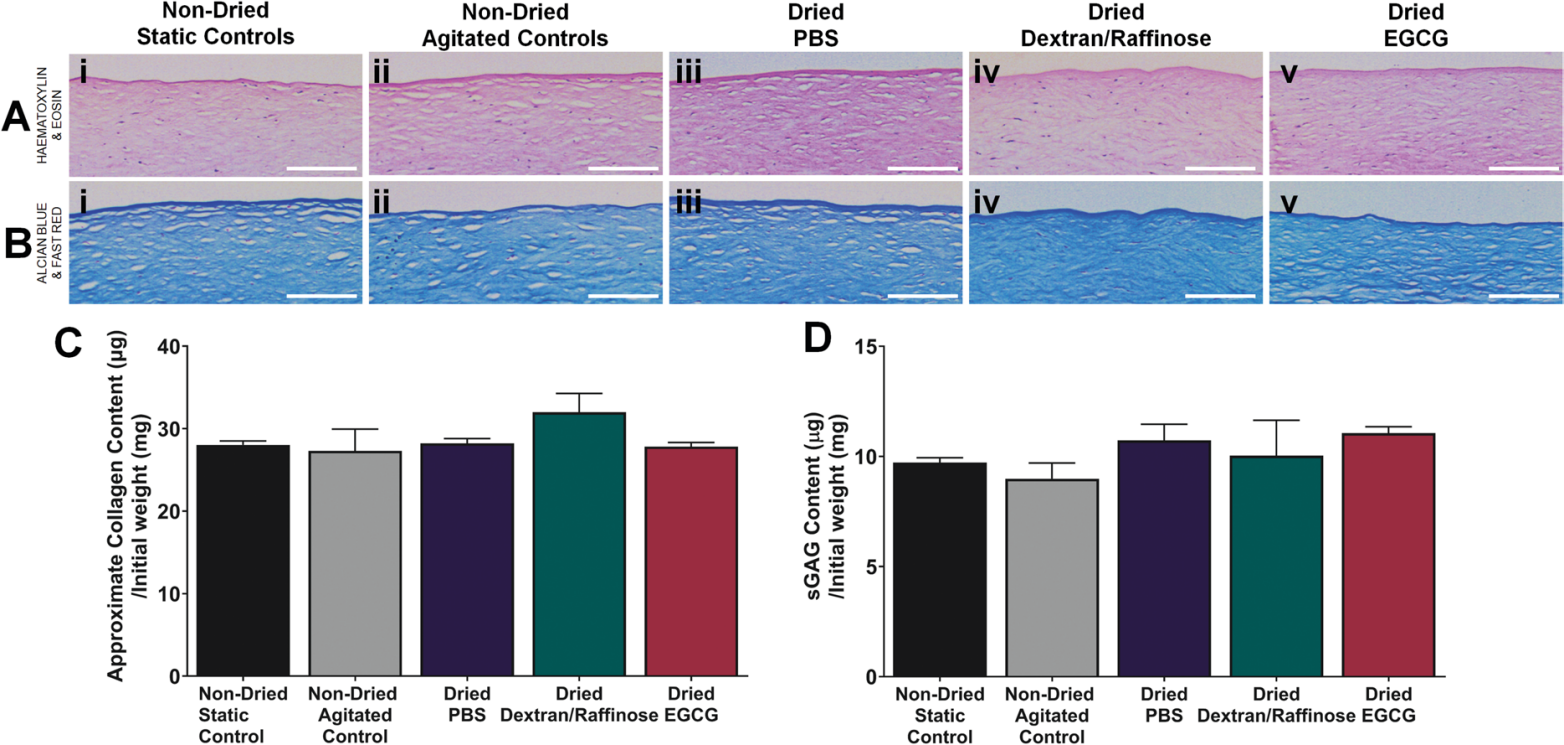
Effect of dry-preservation on collagen and sGAG content of human corneal buttons. Human corneal buttons were dried after agitation in PBS, 5% dextran or 5% dextran with EGCG and compared to non-dried controls that had remained static or were agitated. Dried corneal buttons were rehydrated in NaCl with 5% (w/v) dextran. **A** Representative images of haematoxylin and eosin staining and, **B** alcian blue and fast red staining of sections of treated corneal buttons. Scale bar = 200 µm, **C** Approximate collagen content of corneal buttons with and without drying and rehydration measured by hydroxyproline assay, **D** Approximate sGAG content of corneal buttons with and without drying and rehydration measured by DMMB assay. Data for C and D represented by mean ± SEM (n = 5)

Histological analysis using haematoxylin and eosin staining (Fig. 2A) and alcian blue and fast red staining (Fig. 2B) of corneal buttons revealed no apparent change in anterior stromal architecture compared to non-dried controls. The epithelial and endothelial cells were absent across all structures with minimal disruption of the basement membrane and lamellae structure observed. Quantitative measurements of collagen and sGAG content, using the hydroxyproline assay and DMMB assay respectively, showed no significant loss of collagen or sGAG content between pre-treated and dried buttons and controls. The average collagen content within the samples was maintained at 27 µg/mg collagen and 10 µg/mg sGAG (Fig. 2B and C, respectively.

### Effect of dry-preservation on structure of human corneal buttons

**Fig. 3:**
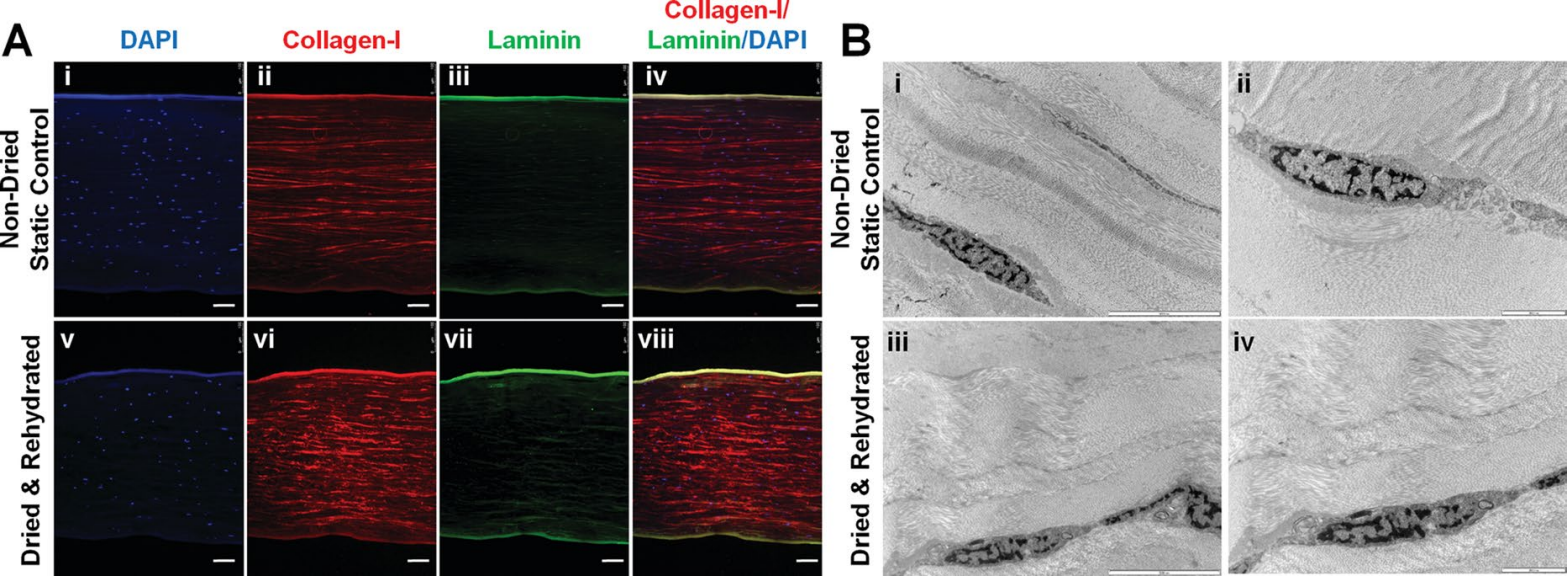
Effect of dry-preservation on structure of human corneal buttons. Human corneal buttons were dried after agitation in 5% dextran and 100 mM raffinose in 0.9% NaCl and compared to static non-dried controls. Dried corneal buttons were rehydrated in NaCl with 5% (w/v) dextran. **A** Representative fluorescent images of sections of human corneas stained via immunohistochemistry for collagen-I and laminin. Scale bar = 100 µm, **B** Representative TEM images of cornea structure pre-drying and after drying and rehydration. Scale bars: i/iii = 5000 nm, ii/iv = 2000 nm

Fluorescent immunohistochemistry (Fig. 3A) of the corneal buttons after drying (dextran/raffinose) and rehydration demonstrated that there was no apparent disruption in collagen-I fibrils or laminin basement membranes. TEM analysis after drying (dextran/raffinose) and rehydration revealed no effect on collagen fibril microstructure (Fig. 3B). Stromal cells were shown to be encapsulated within the collagen fibrils which is comparative with the DAPI staining in the immunohistochemistry.

### Effect of dry-preservation on proteins, growth factors and cytokines within corneal buttons

**Fig. 4:**
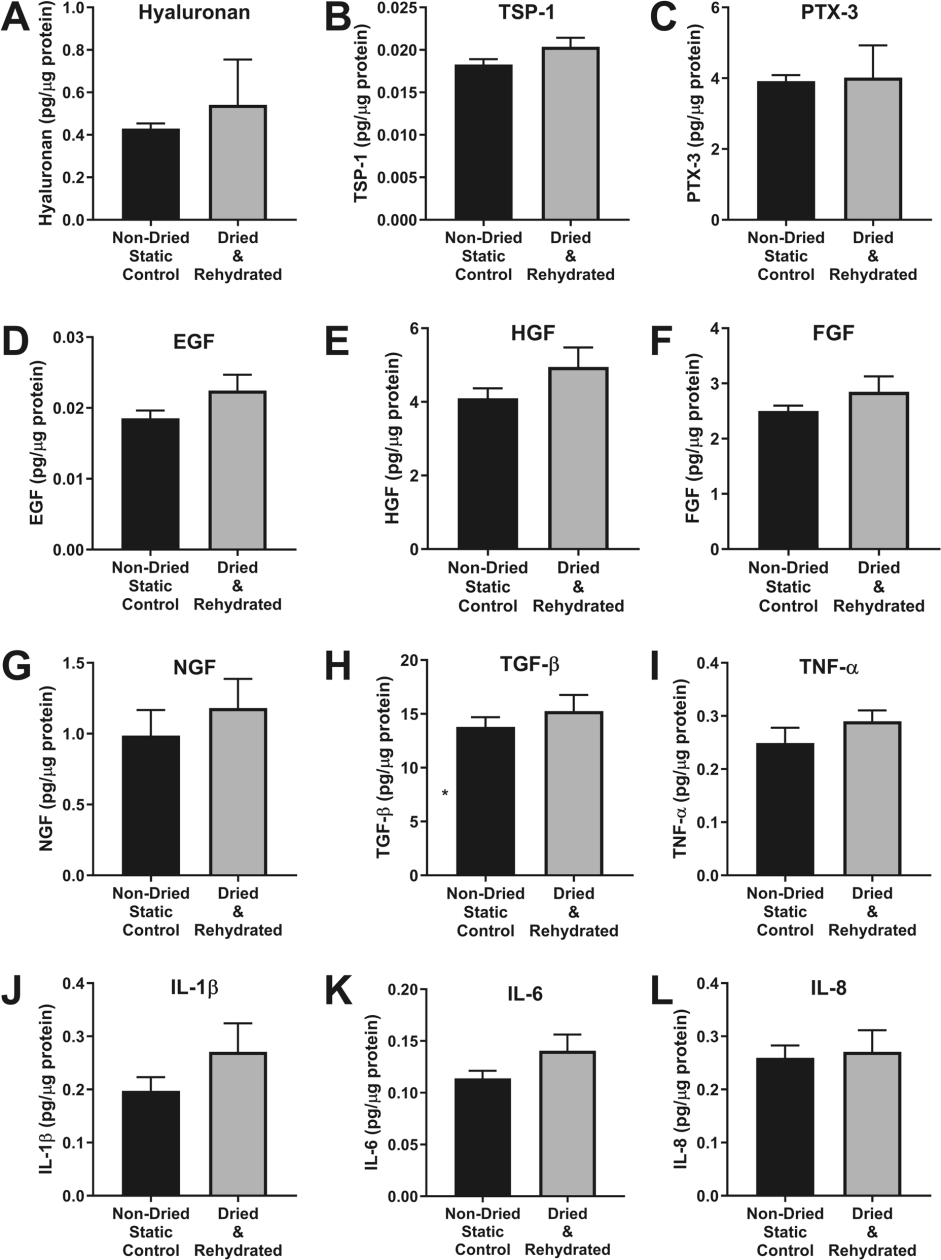
Effect of dry-preservation on proteins, growth factors and cytokines found within corneas. Human corneal buttons were dried after agitation in 5% dextran and 100 mM raffinose in 0.9% NaCl and compared to static non-dried controls. Dried corneal buttons were rehydrated in NaCl with 5% (w/v) dextran and protein was extracted and samples homogenised before ELISAs performed for **A** Hyaluronan, **B** Thrombospondin-1, **C** Pentraxin-3 **D** Epithelial Growth Factor, **E** Hepatocyte Growth Factor, **F** Fibroblast Growth Factor, **G** Nerve Growth Factor, **H** Transforming Growth Factor-β, **I** Tumour Necrosis Factor-α, **J** Interleukin 1-β, **K** Interleukin-6 and **L** Interleukin-8. Date represented by mean ± SEM (n = 5). No statistical significances were found between control and dried

ELISAs were performed for hyaluronan, thrombospondin-1 (TSP-1), pentraxin-3 (PTX-3), epidermal growth factor (EGF), hepatocyte growth factor (HGF), fibroblast growth factor (FGF), nerve growth factor (NGF), transforming growth factor-β (TGF-β), tumour necrosis factor-α (TNF-α), interleukin-1β (IL-1β), interleukin-6 (IL-6) and interleukin-8 (IL-8) (Fig. 4). There were no significant differences in the levels of any assessed protein or sGAG between dry-preserved (dextran/raffinose) and non-dried control corneal buttons.

## Discussion

The adoption of preservation strategies for long-term storage of surplus donor corneal tissue has many benefits: limiting wastage of non-utilised donor corneas; recovery, storage, and use of corneal tissue surplus after surgery; improved global shipping of corneal tissue, including to countries with no eye-banking infrastructure; corneal tissue storage on hospital shelves for emergency use; and alleviating the pressure of a global shortage of donated corneal tissue. The concept of long-term corneal preservation represents a means of delivering a readily available resource for ophthalmologists worldwide, which requires little time and effort for preparation (Gain et al. 2016).

With similarities to lyophilisation but without any freeze-steps, the novel low-temperature vacuum evaporation (LTVE) process, presented in this paper, allows corneal tissue to be stored dry, under vacuum, until needed and rapidly rehydrated prior to clinical use (Sidney et al. 2022). This study demonstrated that this preservation technique has no effect on final corneal tissue weight, transparency and handleability. Using a protective solution of 5% (w/v) dextran and 100 mM raffinose in saline, and subsequently rehydrating in 5% (w/v) dextran in saline, maintains corneal ECM structure and composition, encapsulating the keratocytes within the stromal ECM, rendering them non-viable but not lysed. This study also showed that levels of important functional proteins within the cornea remained unchanged with the application of LTVE and any potential biological effects that these proteins have once transplanted may not be affected by the drying. However, this finding could be expanded by looking into the activity of these proteins and discovering if they are truly functional.

The LTVE process uses gentle agitation to remove the epithelial and endothelial cells without removing the basement membranes, prior to the drying process. The retention of the basement membrane will allow regeneration of the corneal epithelium if used in a lamellar surgery. The removal of the endothelial cells means that the corneal tissue is no longer suitable for full-thickness keratoplasties but could still be utilised in lamellar keratoplasties and stromal transplant surgeries. Using LTVE preserved tissue in lamellar surgeries will ensure that donor corneas with epithelium and endothelium can be used for indications that require them.

LTVE-preserved tissue has flexible usage as it can be cut to size prior to drying or after rehydration by the surgeon. As mentioned previously, the Bowman’s layer and basement membrane of the cornea is still intact so the tissue can be used for lamellar surgeries such as DALKS. The tissue could also be trimmed to stromal lenticules without the basement membranes, allowing use in more modern surgical techniques such as myopic lenticular implant surgery (Semiz et al. 2022) and intrastromal corneal ring segment surgery (Coscarelli et al. 2024) where a smaller stroma-only tissue implant is required. The advantage of using LTVE-preserved tissue for these uses is that non-preserved donor tissue is not used and can be reallocated for full-thickness keratoplasties.

Attempts at long-term preservation of corneal tissue are not a recent phenomenon; the most historically utilised method of long-term storage for corneal tissue is cryopreservation (Brunette et al. 2001; Chen et al. 2010; Eastcott et al. 1954; Halberstadt et al. 2003; Li et al. 2011), which was used clinically prior to the development of 4 °C hypothermic storage. Cryopreservation provided a cheaper alternative with reduced technical demand and variability in its clinical outcome (Eastcott et al. 1954; Halberstadt et al. 2003). The major concern with the cryopreservation procedure is tissue and protein damage due to the formation of ice crystals when freezing (Bojic et al. 2021; Murray et al., 2022). Cooling samples slowly or using cryoprotectants can reduce this effect but in many cases damage to the biological structure and function of the corneal tissue can still be observed, due either to freeze damage or toxicity of the cryoprotectants (Armitage 2009; Bojic et al. 2021). For these reasons cryopreservation is not able to preserve the transparency and functionality of the whole cornea.

Many cryopreservation strategies focus on keeping the cells of the cornea viable, but modern doctrines suggest that this is not required for many corneal indications, as only sections of the cornea will be replaced as in DALK, SALK, patch-grafts and lenticule insertion surgeries, and the host’s own cells can help to repopulate the tissue (Chaurasia et al. 2020). If there is no need to keep the cells viable, dehydration technologies such as LTVE, where the epithelial and endothelial cells are removed and the keratocytes are non-viable, become more attractive.

As a simple technique, glycerol or silica dehydration has been used historically to create dehydrated, storable corneal tissue (King et al. 1961; Romano et al. 2019), however it has also been associated with poor visual outcomes and a high risk of secondary glaucoma (Li et al. 2012; Thanathanee et al. 2016). Unlike corneal tissue dried by LTVE in this study, which can be simply dehydrated in situ approximately 1 h prior to surgery, glycerol corneas must be rehydrated and extensively washed prior to surgical use (King et al., 1984).

The major competitor to our LTVE technique is lyophilisation or freeze drying. Lyophilisation is commonly used across many fields to preserve temperature-sensitive, biological products and is hypothesised to help prevent adverse architectural changes in the cornea (Michael R. Feilmeier et al. 2010; Rovere et al. 2019). Lyophilisation relies on a sublimation process instigated by specific low pressures and increasing temperatures whereby a phase change from solid to vapour occurs, circumventing the liquid stage. Due to the need for the water within the cornea to begin in the solid phase, lyophilisation requires a tissue pre-freeze step. This pre-freeze step, particularly in the absence of cryoprotectants, has been demonstrated to be detrimental to cell integrity and transparency (Lee et al. 2010) and has been associated with causing tissue fragility (Quantock et al. 1997) and a loss of biological proteins (Maini et al. 2020).

LTVE, as described in the study, aims to avoid the disadvantages of the freeze-step in lyophilisation whilst maintaining the advantages of long-term storage and easy distribution and availability (Gain et al. 2016; King et al. 1961). LTVE does not rely on sublimation to remove water from the tissue but instead uniquely uses varying low pressures to accelerate the evaporation rates without application of excessive heat and without allowing the pressure to decrease to the point that the water reaches freezing point (Hackett 2018; Hopkinson et al. 2020). The same technology is used successfully to dry-preserve the amniotic membrane product, Omnigen® (NuVision Biotherapies Ltd, Nottingham, UK) (Claire L. Allen et al. 2013; Hopkinson et al. 2020). The process had been shown to effectively preserve the natural characteristics of tissue, whilst being capable of eradicating an artificially high bacterial load, through a combination of the thorough washing steps and the vacuum-drying process itself (Marsit et al. 2019). The LTVE process for corneas can be performed in a similar aseptic manner to this amniotic membrane processing but still requires full validation to ensure no pathogens are introduced. The process would be relatively easy to introduce into existing eye banks procedures as it requires little specialist equipment other than a freeze-dryer.

Irradiation of corneas is another postulated technique for long-term preservation and can be combined with other methods such as cryopreservation or dehydration. VisionGraft® (CorneaGen, Seattle, WA, USA) is a commercially available inactivated and gamma-irradiated cornea that is suggested to have clinical uses in corneal melts, ulcers, perforations, anterior lamellar keratoplasty, tectonic support and keratoprotheses (Akpek et al. 2012; Mathews et al. 2019; Utine et al. 2011; Wee et al. 2015). The processing for irradiated corneas however still includes a freeze step that still has the potential to cause damage to the structure of the cornea (Kuo 2021) and irradiation of the cornea can also cause changes in the physical and biological composition of the ECM (Chae et al. 2015; Gouk et al. 2008). As this product is stored wet in an albumin solution, it has a shelf life of only 2 years, compared to the estimated 5 years of similar dried products, such as described in our present study, and the transparency of the tissue is reported to decrease with prolonged storage (Kuo 2021). However, the commercial success of gamma-irradiated corneas indicates that there is a large market for long-term preserved corneas and that ophthalmic surgeons are willing to transition to preserved rather than cold-stored or organ culture stored tissue for certain indications. Using long-term preserved corneas for clinical indications for which they are suitable will free-up full-thickness donor corneas for surgeries that require an endothelium or larger amounts of tissue.

In conclusion, the prospect for long-term preserved corneal donor tissue is high and we believe that we have developed a technology that can preserve corneal tissue with minimal disruption to its physical and biological properties. Further investigation needs to be performed on full corneoscleral discs, looking at the functional properties of LTVE treated corneas in a preclinical model but early indications are promising. The full micro and nano-scale effects of the drying process on the ECM structure and composition could also be elucidated further to ensure no lasting effects on corneal function.

A dry-preserved cornea that can potentially be stably stored for years and shipped at room temperature before simple rehydration prior to surgery will allow surplus donor tissue to be fully utilised and the pressure of a global shortage of corneal donor tissue to be alleviated.

## Data Availability

The datasets used and/or analysed during the current study are available from the corresponding author on reasonable request.

## References

[CR1] Akpek EK, Aldave AJ, Aquavella JV (2012) The use of precut, γ-irradiated corneal lenticules in Boston type 1 keratoprosthesis implantation. Am J Ophthalmol 154(3):495–498. 10.1016/j.ajo.2012.03.0224 22633353 10.1016/j.ajo.2012.03.022

[CR2] Allen CL, Clare G, Stewart EA, Branch MJ, McIntosh OD, Dadhwal M, Dua HS, Hopkinson A (2013) Augmented dried versus cryopreserved amniotic membrane as an ocular surface dressing. PLoS ONE 8(10):e78441–e78441. 10.1371/journal.pone.0078441 24205233 10.1371/journal.pone.0078441 PMC3813584

[CR4] Ang M, Moriyama A, Colby K, Sutton G, Liang L, Sharma N, Hjortdal J, Lam SC, D., P Williams, G., Armitage, J., & S Mehta, J. (2020) Corneal transplantation in the aftermath of the COVID-19 pandemic: an international perspective. Br J Ophthalmol 104(11):1477–1481. 10.1136/bjophthalmol-2020-317013 32732343 10.1136/bjophthalmol-2020-317013 PMC7587225

[CR5] Armitage J (2009) Cryopreservation for corneal storage. Dev Ophthalmol 43:63–69. 10.1159/000223839 19494637 10.1159/000223839

[CR6] Armitage WJ (2011) Preservation of Human Cornea. Transfus Med Hemother 38(2):143–147. 10.1159/000326632 21566714 10.1159/000326632 PMC3088736

[CR7] Bojic S, Murray A, Bentley BL, Spindler R, Pawlik P, Cordeiro JL, Bauer R, de Magalhães JP (2021) Winter is coming: the future of cryopreservation. BMC Biol 19(1):56. 10.1186/s12915-021-00976-8 33761937 10.1186/s12915-021-00976-8 PMC7989039

[CR8] Brunette I, Le François M, Tremblay MC, Guertin MC (2001) Corneal transplant tolerance of cryopreservation. Cornea 20(6):590–596. 10.1097/00003226-200108000-00007 11473158 10.1097/00003226-200108000-00007

[CR9] Chae JJ, Choi JS, Lee JD, Lu Q, Stark WJ, Kuo IC, Elisseeff JH (2015) Physical and biological characterization of the gamma-irradiated human cornea. Cornea 34(10):1287–1294. 10.1097/ico.0000000000000555 26203754 10.1097/ICO.0000000000000555

[CR10] Chaurasia S, Das S, Roy A (2020) A review of long-term corneal preservation techniques: relevance and renewed interests in the COVID-19 era. Indian J Ophthalmol 68(7):1357–1363. 10.4103/ijo.IJO_1505_20 32587163 10.4103/ijo.IJO_1505_20 PMC7574093

[CR11] Chen W, Lin Y, Zhang X, Wang L, Liu M, Liu J, Ye Y, Sun L, Ma H, Qu J (2010) Comparison of fresh corneal tissue versus glycerin-cryopreserved corneal tissue in deep anterior lamellar keratoplasty. Invest Ophthalmol vis Sci 51(2):775–781 19737874 10.1167/iovs.09-3422

[CR12] Coscarelli S, Coscarelli SP, Torquetti L (2024) Donut-shaped corneal allogeneic intrastromal segment as an alternative to deep anterior lamellar keratoplasty in advanced keratoconus. Cornea 43(5):658–663. 10.1097/ico.0000000000003456 38178305 10.1097/ICO.0000000000003456

[CR13] Eastcott HH, Cross AG, Leigh AG, North DP (1954) Preservation of corneal grafts by freezing. Lancet 266(6805):237–239. 10.1016/s0140-6736(54)90879-x 13131830 10.1016/s0140-6736(54)90879-x

[CR14] Edwards CA, O’Brien WD Jr (1980) Modified assay for determination of hydroxyproline in a tissue hydrolyzate. Clin Chim Acta 104(2):161–167 7389130 10.1016/0009-8981(80)90192-8

[CR15] Feilmeier MR, Tabin GC, Williams L, Oliva M (2010) The use of glycerol-preserved corneas in the developing world. Middle East Afr J Ophthalmol 17(1):38–43. 10.4103/0974-9233.61215 20543935 10.4103/0974-9233.61215 PMC2880372

[CR17] Gain P, Jullienne R, He Z, Aldossary M, Acquart S, Cognasse F, Thuret G (2016) Global survey of corneal transplantation and eye banking. JAMA Ophthalmol 134(2):167–173. 10.1001/jamaophthalmol.2015.4776 26633035 10.1001/jamaophthalmol.2015.4776

[CR18] Ganger A, Tandon R, Vanathi M, Sagar P (2016) Superficial anterior lamellar keratoplasty (SALK) for trauma-induced post refractive surgery corneal opacity. J Ophthalmic Vis Res 11(3):326–328. 10.4103/2008-322x.188394 27621794 10.4103/2008-322X.188394 PMC5000539

[CR19] Gouk SS, Lim TM, Teoh SH, Sun WQ (2008) Alterations of human acellular tissue matrix by gamma irradiation: histology, biomechanical property, stability, in vitro cell repopulation, and remodeling. J Biomed Mater Res B Appl Biomater 84(1):205–217. 10.1002/jbm.b.30862 17497685 10.1002/jbm.b.30862

[CR20] Hackett BW (2018) The essentials of continuous evaporation American Institute of Chemical Engineers.

[CR21] Halberstadt M, Böhnke M, Athmann S, Hagenah M (2003) Cryopreservation of human donor corneas with dextran. Invest Ophthalmol vis Sci 44(12):5110–5115. 10.1167/iovs.03-0370 14638705 10.1167/iovs.03-0370

[CR22] Hopkinson A, Britchford ER, Sidney LE (2020) Preparation of dried amniotic membrane for corneal repair. Methods Mol Biol 2145:143–157. 10.1007/978-1-0716-0599-8_10 32542605 10.1007/978-1-0716-0599-8_10

[CR23] Karimian F, Feizi S (2010) Deep anterior lamellar keratoplasty: indications, surgical techniques and complications. Middle East Afr J Ophthalmol 17(1):28–37. 10.4103/0974-9233.61214 20543934 10.4103/0974-9233.61214 PMC2880371

[CR24] Keenan TD, Jones MN, Rushton S, Carley FM (2012) Trends in the indications for corneal graft surgery in the United Kingdom: 1999 through 2009. Arch Ophthalmol 130(5):621–628. 10.1001/archophthalmol.2011.2585 22652847 10.1001/archophthalmol.2011.2585

[CR25] King JH Jr, Townsend WM (1984) The prolonged storage of donor corneas by glycerine dehydration. Trans Am Ophthalmol Soc 82:106–110 6398932 PMC1298657

[CR26] King JH Jr, Mc TJ, Meryman HT (1961) Preservation of corneas for lamellar keratoplasty: a simple method of chemical glycerine-dehydration. Trans Am Ophthalmol Soc 59:194–201 14456106 PMC1316406

[CR27] Kuo IC (2021) Review of gamma-irradiated sterile cornea: properties, indications, and new directions. Eye Contact Lens 47(4):157–162. 10.1097/icl.0000000000000722 32568928 10.1097/ICL.0000000000000722

[CR28] Lee JK, Ryu YH, Ahn JI, Kim MK, Lee TS, Kim JC (2010) The effect of lyophilization on graft acceptance in experimental xenotransplantation using porcine cornea. Artif Organs 34(1):37–45. 10.1111/j.1525-1594.2009.00789.x 19821814 10.1111/j.1525-1594.2009.00789.x

[CR29] Li J, Yu L, Deng Z, Wang L, Sun L, Ma H, Chen W (2011) Deep anterior lamellar keratoplasty using acellular corneal tissue for prevention of allograft rejection in high-risk corneas. Am J Ophthalmol 152(5):762–770 21803324 10.1016/j.ajo.2011.05.002

[CR30] Li J, Shi S, Zhang X, Ni S, Wang Y, Curcio CA, Chen W (2012) Comparison of different methods of glycerol preservation for deep anterior lamellar keratoplasty eligible corneas. Invest Ophthalmol Vis Sci 53(9):5675–5685. 10.1167/iovs.12-9936 22836770 10.1167/iovs.12-9936

[CR31] Lim CHL, Riau AK, Lwin NC, Chaurasia SS, Tan DT, Mehta JS (2013) LASIK following small incision lenticule extraction (SMILE) Lenticule Re-Implantation: a feasibility study of a novel method for treatment of presbyopia. PLoS ONE 8(12):e83046. 10.1371/journal.pone.0083046 24349429 10.1371/journal.pone.0083046 PMC3859649

[CR32] Lindstrom RL, Kaufman HE, Skelnik DL, Laing RA, Lass JH, Musch DC, Trousdale MD, Reinhart WJ, Burris TE, Sugar A et al (1992) Optisol corneal storage medium. Am J Ophthalmol 114(3):345–356. 10.1016/s0002-9394(14)71803-3 1524127 10.1016/s0002-9394(14)71803-3

[CR33] Lynch AP, Ahearne M (2013) Mar). Strategies for developing decellularized corneal scaffolds. Exp Eye Res 108:42–47. 10.1016/j.exer.2012.12.012 23287438 10.1016/j.exer.2012.12.012

[CR34] Lynch AP, Wilson SL, Ahearne M (2016) Dextran preserves native corneal structure during decellularization. Tissue Eng Part C Methods 22(6):561–572. 10.1089/ten.TEC.2016.0017 27068608 10.1089/ten.TEC.2016.0017

[CR35] Machin H, Arslan J, Baird PN (2020) Examining the impact of corneal tissue transnational activity, and transplantation, on import and export nations: a review of the literature. Cornea 39(6):795–800. 10.1097/ico.0000000000002255 31939918 10.1097/ICO.0000000000002255

[CR36] Maini, S., Hurley-Bennett, K., & Dawson, C. (2020, 2020/11/01/). Case Series Describing the Use of Low-Temperature Vacuum-Dehydrated Amnion (Omnigen) for the Treatment of Corneal Ulcers in Cats and Dogs: 46 Cases (2016–2017). *Topics in Companion Animal Medicine, 41*, 100474. Doi;10.1016/j.tcam.2020.100474 10.1016/j.tcam.2020.100474 32919060

[CR37] Manche EE, Holland GN, Maloney RK (1999) Deep lamellar keratoplasty using viscoelastic dissection. Arch Ophthalmol 117(11):1561–1565. 10.1001/archopht.117.11.1561 10565532 10.1001/archopht.117.11.1561

[CR38] Marsit NM, Sidney LE, Britchford ER, McIntosh OD, Allen CL, Ashraf W, Bayston R, Hopkinson A (2019) Validation and assessment of an antibiotic-based, aseptic decontamination manufacturing protocol for therapeutic, vacuum-dried human amniotic membrane. Sci Rep 9(1):12854. 10.1038/s41598-019-49314-7 31492886 10.1038/s41598-019-49314-7 PMC6731261

[CR39] Mathews PM, Fogla R, Samayoa E, VanCourt S, Akpek EK (2019) Long-term clinical outcomes of keratoplasty using gamma-irradiated corneal lenticules. BMJ Open Ophthalmol 4(1):e000396. 10.1136/bmjophth-2019-000396 31799412 10.1136/bmjophth-2019-000396 PMC6861079

[CR40] Moshirfar M, Shah TJ, Masud M, Fanning T, Linn SH, Ronquillo Y, Hoopes PCS (2018) A modified small-incision lenticule intrastromal keratoplasty (sLIKE) for the correction of high hyperopia: a description of a new surgical technique and comparison to lenticule intrastromal keratoplasty (LIKE). Med Hypothesis Discov Innov Ophthalmol 7(2):48–56 30250852 PMC6146242

[CR41] Murray KA, Gibson MI (2022) Chemical approaches to cryopreservation. Nat Rev Chem 6(8):579–593. 10.1038/s41570-022-00407-4 35875681 10.1038/s41570-022-00407-4 PMC9294745

[CR42] Natan D, Nagler A, Arav A (2009) Freeze-drying of mononuclear cells derived from umbilical cord blood followed by colony formation. PLoS ONE 4(4):e5240. 10.1371/journal.pone.0005240 19381290 10.1371/journal.pone.0005240 PMC2667668

[CR43] Oliva J, Florentino A, Bardag-Gorce F, Niihara Y (2019) Vitrification and storage of oral mucosa epithelial cell sheets. J Tissue Eng Regen Med 13(7):1153–1163. 10.1002/term.2864 30964962 10.1002/term.2864 PMC6767061

[CR44] Pels L (1997) Organ culture: the method of choice for preservation of human donor corneas. Br J Ophthalmol 81(7):523. 10.1136/bjo.81.7.523 9290360 10.1136/bjo.81.7.523 PMC1722261

[CR45] Peyman GA, Sanders DR, Ligara TH (1979) Dextran 40-containing infusion fluids and corneal swelling: a specular microscopic study. Arch Ophthalmol 97(1):152–155. 10.1001/archopht.1979.01020010086020 758892 10.1001/archopht.1979.01020010086020

[CR46] Quantock AJ, Verity SM, Schanzlin DJ (1997) Organization of collagen in the lyophilized cornea. J Refract Surg 13(2):167–170 9109074 10.3928/1081-597X-19970301-14

[CR47] Romano V, Levis HJ, Gallon P, Lace R, Borroni D, Ponzin D, Ruzza A, Kaye SB, Ferrari S, Parekh M (2019) Biobanking of dehydrated human donor corneal stroma to increase the supply of anterior lamellar grafts. Cornea 38(4):480–484. 10.1097/ico.0000000000001876 30681513 10.1097/ICO.0000000000001876

[CR48] Rovere MR, Ouilhon C, Salmon D, Haftek M, Damour O, Auxenfans C (2019) Development and characterization of lyophilized transparized decellularized stroma as a replacement for living cornea in deep anterior lamellar keratoplasty. Cell Tissue Bank 20(1):49–59. 10.1007/s10561-018-9742-x 30719600 10.1007/s10561-018-9742-x

[CR49] Semiz F, Lokaj AS, Tanriverdi G, Caliskan G, Hima-Musa N, Semiz CE (2022) Fresh human myopic lenticule intrastromal implantation for keratoconus using SMILE surgery in a long-term follow-up study: ultrastructural analysis by transmission electron microscopy. J Refract Surg 38(8):520–528. 10.3928/1081597x-20220713-02 35947000 10.3928/1081597X-20220713-02

[CR50] Sidney LE, Hopkinson A, McIntosh OD (2022) Corneal Tissue. European Patent Office,EP4057940A1;US2022387168A1;WO2021094780A1.

[CR51] Sidney LE, Hopkinson A (2018) Corneal keratocyte transition to mesenchymal stem cell phenotype and reversal using serum-free medium supplemented with fibroblast growth factor-2, transforming growth factor-beta3 and retinoic acid. J Tissue Eng Regen Med 12(1):e203–e215. 10.1002/term.2316 27685949 10.1002/term.2316

[CR52] Thanathanee O, Sripawadkul W, Anutarapongpan O, Luanratanakorn P, Suwan-Apichon O (2016) Outcome of therapeutic penetrating keratoplasty using glycerol-preserved donor corneas in infectious keratitis. Cornea. 10.1097/ICO.0000000000000841 27429078 10.1097/ICO.0000000000000841

[CR53] Thirunavukarasu AJ, Han E, Nedumaran AM, Kurz AC, Shuman J, Yusoff N, Liu YC, Foo V, Czarny B, Riau AK, Mehta JS (2023) Oct 1). Electron beam-irradiated donor cornea for on-demand lenticule implantation to treat corneal diseases and refractive error. Acta Biomater 169:334–347. 10.1016/j.actbio.2023.07.053 37532130 10.1016/j.actbio.2023.07.053

[CR54] Utine, C. A., Tzu, J. H., & Akpek, E. K. (2011). Lamellar keratoplasty using gamma-irradiated corneal lenticules. *Am J Ophthalmol, 151*(1), 170–174.e171. 10.1016/j.ajo.2010.08.007 10.1016/j.ajo.2010.08.007 21145036

[CR55] Wee, S. W., Choi, S. U., & Kim, J. C. (2015, Apr). Deep anterior lamellar keratoplasty using irradiated acellular cornea with amniotic membrane transplantation for intractable ocular surface diseases. *Korean J Ophthalmol, 29*(2), 79–85. 10.3341/kjo.2015.29.2.79 10.3341/kjo.2015.29.2.79 PMC4369521 25829823

[CR56] Wojcik, G., Ferrari, S., Romano, V., Ponzin, D., Ahmad, S., & Parekh, M. (2021, 2021/01/02). Corneal storage methods: considerations and impact on surgical outcomes. *Expert Review of Ophthalmology, 16*(1), 1–9. 10.1080/17469899.2021.1829476

[CR57] Zaki AA, Elalfy MS, Said DG, Dua HS (2015) Deep anterior lamellar keratoplasty–triple procedure: a useful clinical application of the pre-Descemet’s layer (Dua’s layer). Eye (Lond) 29(3):323–326. 10.1038/eye.2014.273 25359285 10.1038/eye.2014.273 PMC4366456

